# Cardiac Relapse of Acute Myeloid Leukemia after Allogeneic Hematopoietic Stem Cell Transplantation

**DOI:** 10.1155/2016/5091021

**Published:** 2016-08-23

**Authors:** María Facenda-Lorenzo, Ana Sánchez-Quintana, Alejandro Quijada-Fumero, Ana Laynez-Carnicero, Joaquín Breña-Atienza, Francisco J. Poncela-Mireles, Juan M. Llanos-Gómez, Ana I. Cabello-Rodríguez, María Ramos-López

**Affiliations:** ^1^Servicio de Cardiología, Hospital Universitario de La Candelaria, Carretera del Rosario No. 145, 38010 Santa Cruz de Tenerife, Spain; ^2^Hematology Service, University Hospital Nuestra Señora de La Candelaria, Santa Cruz de Tenerife, Tenerife, Canarias, Spain; ^3^Radiology Service, University Hospital Nuestra Señora de La Candelaria, Santa Cruz de Tenerife, Tenerife, Canarias, Spain

## Abstract

Secondary or metastatic cardiac tumors are much more common than primary benign or malignant cardiac tumors. Any tumor can cause myocardial or pericardial metastasis, although isolated or combined tumor invasion of the pericardium is more common. Types of neoplasia with the highest rates of cardiac or pericardial involvement are melanoma, lung cancer, and breast and mediastinal carcinomas. Acute myeloid leukemia (AML) is the most common type of acute leukemia in adults. Initial treatment involves chemotherapy followed by consolidation treatment to reduce the risk of relapse. In high-risk patients, the treatment of choice for consolidation is hematopoietic stem cell transplantation (HSCT). Relapse of AML is the most common cause of HSCT failure. Extramedullary relapse is rare. The organs most frequently affected, called “sanctuaries,” are the testes, ovaries, and central nervous system. We present a case with extramedullary relapse in the form of a solid cardiac mass.

## 1. Introduction

Secondary or metastatic cardiac tumors are much more common than primary benign or malignant cardiac tumors. They usually appear after the age of 50 years in patients of either sex [1]. Metastasis to the heart can occur by direct invasion (mediastinal tumors), hematogenous spread, lymphatic spread, and intracavitary extension through the inferior vena cava [2]. Any tumor can cause myocardial or pericardial metastasis, although isolated or combined tumor invasion of the pericardium is more common. Types of neoplasia with the highest rates of heart or pericardial involvement are melanoma, lung cancer, breast carcinoma, and mediastinal carcinomas [3]. Clinical presentation depends primarily on the size of the tumor and its anatomical location rather than histological type. Thus, the symptoms can be grouped into three main categories: pericardial involvement can produce pericardial effusion or, less frequently, pericarditis, and myocardial involvement is associated with atrial or ventricular arrhythmias, with varying degrees of blockages and angina due to compression or tumor embolization, while right intracavitary tumors can cause right heart occupation [4]. Transthoracic echocardiography (TTE) is the primary diagnostic procedure, followed by computed tomography (CT) and cardiac magnetic resonance imaging (MRI) to verify positive findings and to analyze other structures of the mediastinum and chest [1]. The final diagnosis requires histologic confirmation. Treatment of such tumors is generally associated with poor results and unfavorable prognosis [1].

Acute myeloid leukemia (AML) is a hematologic malignancy caused by the clonal transformation of a cell of myeloid lineage which proliferates abnormally, leading to the accumulation of immature myeloid cells in bone marrow (BM) and blood. AML is the most common type of acute leukemia in adults and its incidence increases with age [5]. In young patients, AML is a potentially curable disease. Initial treatment involves chemotherapy, aimed at inducing remission of cancer cells, followed by consolidation treatment to reduce the risk of relapse. In high-risk patients the treatment of choice for consolidation is hematopoietic stem cell transplantation (HSCT) from a compatible donor [5].

Recurrence of AML is the most common cause of HSCT treatment failure, occurring in up to 40% of patients who are refractory to first-line treatment. Extramedullary relapse is considered rare although its reported incidence varies from 0.65% to more than 20% [6]. AML may be located at a single site or manifest diffusely with multiorgan involvement. The organs most often affected, called “sanctuaries,” are the testes, ovaries, and central nervous system, but bones, paranasal sinuses, breast tissue, skin, gastrointestinal tract, and the kidneys can also be affected [6, 7].

## 2. Case Presentation

We report the case of a 61-year-old woman diagnosed with AML without maturation in September 2013. Conventional cytogenetic analysis revealed 46,XX. PCR analyses did not reveal NPM1 or FLT3/ITD mutation. She received induction chemotherapy with daunorubicin and cytarabine with primary refractoriness and reinduction chemotherapy with fludarabine, cytarabine, and idarubicin (FLAG-IDA) with which complete remission was achieved. Consolidation was performed with allogeneic matched-sibling HSCT in January 2014, with thiotepa, fludarabine, and busulfan conditioning. Posttransplant complications presented as cytomegalovirus infection and acute gastric graft versus host disease (GVHD). One year after HSCT, immunosuppression (IS) was withdrawn, after which the patient presented chronic GVHD of the liver and eyes, which required the reintroduction of IS with sirolimus and corticosteroids. As part of follow-up, TTE performed in October 2014 showed no evidence of structural or valvular heart disease.

**Figure 1:**
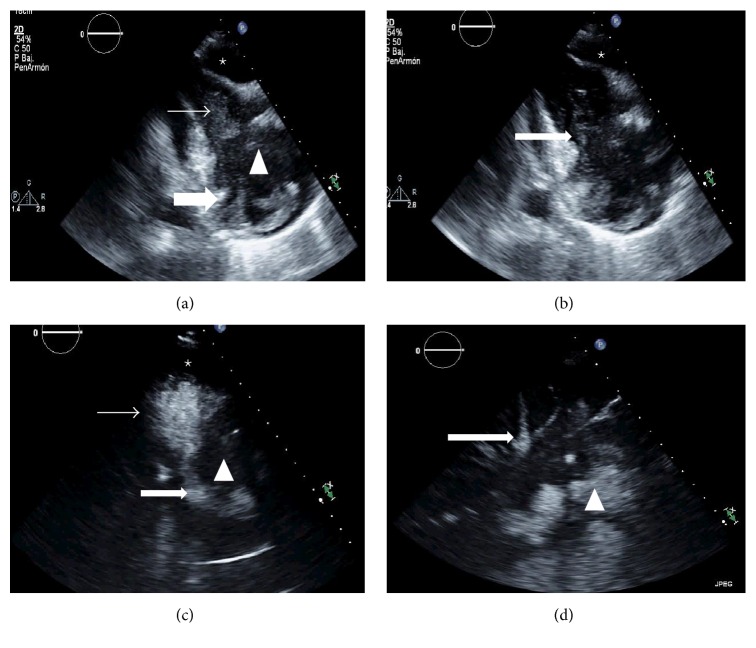
Transthoracic echocardiography with SonoVue® contrast enhancement: (a) shows a homogeneous mass (arrowhead) in the right atrium (RA, thick arrow) which hindered blood flow to the right ventricle (RV, thin arrow), containing contrast medium. Pericardial effusion (star). (b) Same plane image showing stenosis at the tricuspid valve (arrow), with some contrast passing to the RV, as well as tricuspid stenosis. Pericardial effusion (star). (d) Echocardiogram showing contrast in most of the RV (thin arrow) and RA (thick arrow). The arrowhead refers to the homogeneous atrial mass. Pericardial effusion (star). (e) Subcostal plane, showing the contrast (arrowhead) and reflux into the suprahepatic veins (arrow).

**Figure 2:**
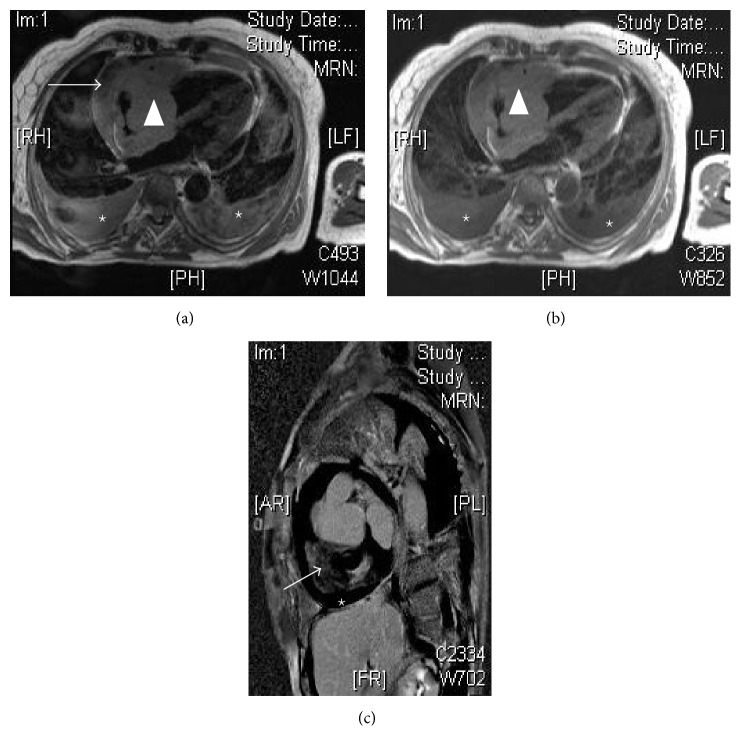
Cardiac magnetic resonance imaging (MRI). (a) Axial T2 cardiac section, showing an isointense homogeneous mass with respect to the RA myocardium (arrowhead), with pericardial effusion (arrow) and bilateral pleural effusion (star). (b) Axial T1 section, similar to the previous image, showing an isointense homogeneous mass with respect to the myocardium that occupies almost all of the RA, without fat infiltration (arrowhead) and bilateral pleural effusion (star). (c) shows the short-axis RV with delayed gadolinium enhancement and a pattern of diffuse and ill-defined enhancement (arrow). Pericardial effusion (star).

In August 2015 the patient was admitted to the emergency department with symptoms of malaise, fever, and pain characteristic of pericarditis. Physical examination revealed a blood pressure of 120/60 mmHg and mild 45-degree jugular venous distension. Cardiopulmonary auscultation showed decreased vesicular murmur in bases with symptoms of pleural effusion, no added noise and arrhythmic low-pitched heart sounds, and slight lower limb edemas. The electrocardiogram showed atrial fibrillation with controlled ventricular voltages and low generalized response. TTE showed left cavities of normal size and function and a dilated right ventricle (RV) with a mass of maximum diameter of 30 mm in the right atrium (RA) that hindered blood flow from the RA to the RV (mean tricuspid gradient of 8 mmHg). TTE with contrast (SonoVue) showed thickened, rigid RA walls and an anchored mass measuring 30 × 33 mm, seemingly dependent on the interatrial septum, extending to the lateral side of the RV, and moderate pericardial effusion (anterior 10 mm, posterior 8 mm, and RV free wall 13 mm) without evidence of hemodynamic compromise (Figures 1(a)–1(d)). Cardiac MRI (Figures 2(a)–2(c)) showed a mass with irregular edges occupying almost the whole RA, with concentric wall involvement and extension to basal segments of the RV free wall, encompassing the right coronary artery without causing stenosis, measuring 53 × 62 mm on the 4-chamber axis. The mass was homogeneous, isointensewith respect to the myocardium, without presenting fatty infiltration or hypervascularization on perfusion sequence, with a diffuse and delayed enhancement pattern, ill-defined, and non-specific, as well as moderate-severe pericardial effusion and bilateral pleural effusion. Thoracic-abdominal-pelvic CT scan showed a RA mass extending to the RV, as well as pleural and pericardial effusion.

**Figure 3:**
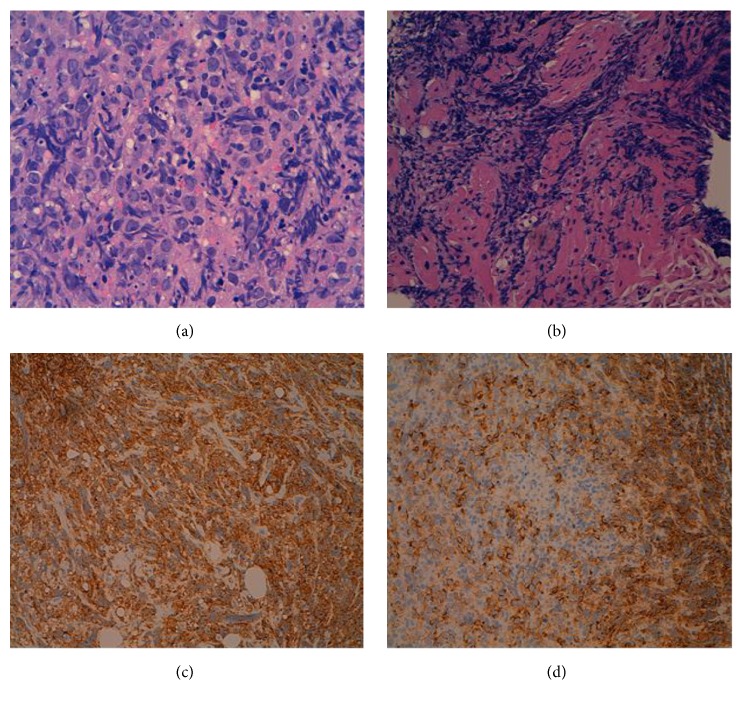
Histological findings. ((a) and (b)) The endomyocardial biopsy showed blastic infiltrate associated with myocardial necrosis (hematoxylin and eosin stain). ((c) and (d)) Infiltrating cells were strongly positive for CD45 (c) and CD34 (d).

Atrial mass biopsy (Figures 3(a)–3(d)) showed fragments of necrotic tissue with groups of viable blast-like cells, compatible with morphologic and immunophenotypic leukemic infiltration. In addition, BM aspirate confirmed the presence of relapse. After reinduction with FLAG-IDA, infectious and cardiologic complications appeared in the form of slow atrial fibrillation, which was treated with isoproterenol. Finally the patient died 10 days after reinitiating chemotherapy.

## 3. Discussion

Previous publications have reported that leukemia can metastasize to the heart [1, 8], usually presenting as diffuse infiltration of the pericardium and myocardium [9]. Also, extramedullary relapse after HSCT is known to occur in some patients, but usually later than isolated bone marrow relapses (mean 13 versus 6 months after HSCT). Most of these patients have a history of chronic GVHD and only a minority have extramedullary disease at diagnosis. The novelty of the present case is that the relapse manifested as a solid heart mass, which is exceptional in AML. On reviewing the literature we found very few cases of cardiac relapse after HSCT and mainly in acute lymphoblastic leukemia [10–13]. However, we did find one case of pericardial involvement after BM transplantation for AML [14].

The present case once again illustrates the importance of imaging techniques and biopsy to confirm the diagnosis of any cardiac mass, taking into account neoplasms in the differential diagnosis. It also illustrates that extramedullary relapse after BM transplant for AML can, exceptionally, take the form of a solid cardiac tumor. To our knowledge, this has not been previously reported.
